# Effectiveness of Psychoeducation via Telenursing on Reducing Caregiver Burden Among Caregivers for Patients with Schizophrenia in Saudi Arabia: A Quasi-Experimental Study

**DOI:** 10.3390/healthcare13151922

**Published:** 2025-08-06

**Authors:** Loujain Sharif, Manal Sadan Al-Zahrani, Fatimah Raji Alanzi, Alaa Mahsoon, Khalid Sharif, Sultan Ahmed Al-Qubali, Rebecca J. Wright, Ayman Mohamed El-Ashry

**Affiliations:** 1 Psychiatric and Mental Health Nursing, Faculty of Nursing, King Abdulaziz University, Jeddah 21551, Saudi Arabia; mahsoon@kau.edu.sa; 2 Psychiatric and Mental Health Nursing, Tabuk Health Cluster, Tabuk 71421, Saudi Arabia; malzahrani37@moh.gov.sa; 3 Psychiatric and Mental Health Nursing, Al Madinah Health Cluster, Madinah 42311, Saudi Arabia; fatimahra@moh.gov.sa; 4 Department of Behavioral Medicine and Psychiatry, West Virginia University, Morgantown, WV 26505, USA; khalid.sharif.md@gmail.com; 5 Surgery and Internal Medicine, Tabuk Health Cluster, Tabuk 47311, Saudi Arabia; salqubali@moh.gov.sa; 6 School of Nursing, Johns Hopkins University, Baltimore, MD 21205, USA; rebecca.wright@jhu.edu; 7 Psychiatric and Mental Health Nursing, Faculty of Nursing, Alexandria University, Alexandria 21527, Egypt; amelashry@ju.edu.sa; 8 Psychiatric and Mental Health Nursing, Nursing Department, College of Applied Medical Sciences, Jouf University, Al-Qurayyat 72388, Saudi Arabia

**Keywords:** psychoeducation, telenursing, caregivers, burden, caring, patients, schizophrenia, Saudi Arabia, quasi-experimental study

## Abstract

**Background/Objectives**: Family caregivers of individuals with schizophrenia often face considerable psychological and physical strain due to the complexity of caregiving. Although psychoeducation has demonstrated benefits in alleviating this burden, its provision via telenursing remains underexplored in Saudi Arabia. This study evaluated the effect of a psychoeducational program delivered via telenursing on reducing caregiver burden. **Methods**: A quasi-experimental design was used with 60 caregivers from a tertiary mental health hospital in northern Saudi Arabia, who were divided equally into intervention and control groups. The intervention group participated in a structured four-week psychoeducational program via Zoom, while the control group received routine care. Caregiver burden was assessed using the Family Burden Interview Schedule (FBIS), a validated tool designed to measure the objective and subjective burden experienced by family members caring for individuals with mental illness. The FBIS was administered before and three months after the intervention. The statistical analysis included independent and paired *t*-tests and ANOVA. **Results**: The pre-intervention scores showed no significant differences, confirming baseline equivalence. The post-intervention scores showed a significant reduction in burden among the intervention group (*p* < 0.001), while no meaningful change occurred in the control group. Additionally, a lower burden was associated with higher education, sufficient income (i.e., the caregiver’s perception of being able to meet essential household expenses without financial strain), strong family support, and absence of caregiver illness. **Conclusions**: These findings suggest that psychoeducation through telenursing is an effective strategy for reducing caregiver burden and improving support accessibility, particularly for those in remote areas.

## 1. Introduction

A caregiver is a person responsible for taking care of a patient at the latter’s place of residence and tasked with implementing the instructions given by the medical health team [1]. Family caregivers encounter various obstacles when caring for a family member with a mental illness, such as a lack of training and basic knowledge about mental illness, and social distress, which often manifests due to social stigma [2]. Caregivers are often considered a forgotten link in the mental health care continuum because their essential contributions—such as managing daily care, promoting treatment adherence, and providing emotional and logistical support—are frequently overlooked in formal healthcare planning, training, and decision-making processes. Therefore, the pressure they face in providing care for mental health patients makes them vulnerable to stress, anxiety, social isolation, and burnout [3]. Home caregivers’ roles vary from aiding patients with daily activities to implementing treatment plans. In addition, family caregivers empower patients to become more independent by supporting adherence to treatment. Moreover, the role of caregivers has expanded over time as they are now responsible for executing medical and nursing guidelines and providing care after patients are discharged from mental health facilities [4,5].

A strong relationship between caregivers of patients with mental illnesses and the healthcare system leads to improved quality of life for both families and patients, which in turn shortens the length of hospital stays and can delay or prevent psychiatric readmissions by promoting stability and medication adherence at home [6,7].

Conversely, poor connections between families and the healthcare system negatively impact both patients—by contributing to treatment nonadherence, relapse, and hospital readmission—and caregivers, who may feel unsupported and ill-equipped to effectively manage care at home [8]. Notably, studies have found that caregivers experience care burden and stress after patients are discharged from the hospital [9,10].

Currently, education is an essential part of the nursing profession, which can enhance the quality of life for both patients and caregivers. Moreover, using an educational approach appears to be an effective method for preventing care overload and disease complications [11]. Psychoeducation is defined as the provision of education and guidance to patients with mental illness and their caregivers regarding all necessary information about predisposing factors, etiology, prognosis, treatment modalities and alternatives, and possible complications and side effects [12]. Psychoeducation can be delivered individually or occur in group therapy sessions moderated by qualified healthcare professionals, such as psychiatric nurses, psychologists, social workers, and physicians [13]. The goal of psychoeducation is to provide essential knowledge and information to patients and their caregivers about the illness, ensure patient adherence to treatment plans, prevent disease relapse, and offer counseling regarding suicide prevention and crisis management [12].

Telenursing is defined as the provision of nursing care using communication technologies, such as telephone, internet, or virtual clinics, to save time and provide better care for caregivers who live far from mental health facilities [14]. Telenursing is a useful method for monitoring patient progress after hospital discharge and for addressing potential gaps in care [15]. Studies have found that telenursing has positive effects, such as decreasing anxiety, alcohol use, and caregiver burden among caregivers of patients with schizophrenia who have limited access to healthcare services [16,17]. Notably, many studies have reported that telenursing positively impacts mental health through multiple mechanisms including increased medication adherence in patients with schizophrenia [18,19,20,21,22].

Schizophrenia is a serious mental illness associated with psychosis and disability that affects patients’ lives, causes deterioration in personal, social, and occupational functioning, increases family frustration, and places significant strain on patient caregivers [23]. Schizophrenia typically occurs in late adolescence, with its onset generally occurring earlier in males than females. It affects approximately 24 million individuals globally [24].

Despite a wealth of studies on psychoeducation delivered through telenursing in healthcare, little is known about its effectiveness in reducing the burden of care among caregivers of patients with schizophrenia. Historically, professionals have created educational interventions for patients and caregivers; however, increasing evidence suggests that such interventions are more likely to be accepted by their intended recipients when their development includes some form of collaboration or co-design [25]. Consequently, many studies have confirmed the effectiveness of telenursing psychoeducation sessions in managing family stress, enhancing family engagement in treatment plans, increasing family life satisfaction, and improving social contribution, all of which contribute to a reduction in caregiver burden [16].

Despite promising outcomes, tele-psychoeducation and telehealth interventions have yielded mixed evidence regarding both clinical effectiveness (e.g., caregiver burden and quality of life) and economic sustainability. Systematic reviews suggest that remote chronic-disease management can improve patient quality of life, but inconsistent effects on mental health outcomes persist [26]. Meanwhile, cost-effectiveness analyses—such as the Barwon PHC trial (COPD/diabetes)—have shown potential health gains (~0.09 QALY) with modest additional costs, but the significance depends on the scale and follow-up duration [27].

In Saudi Arabia, few studies have examined the importance of psychoeducation delivered through telenursing in healthcare, although telenursing has been found to be an effective education technique because it promotes continuity of care for patients and supports informal caregivers [28]. Notably, applying education through telemedicine in Saudia Arabia is important, as it adopts a patient-centered approach that can be cost-effective, easily connecting patients and caregivers to medical services, and reducing health disparities and patient waiting times [29].

The current experimental study was conducted to address this research gap, since, to the authors’ knowledge, no experimental study in Saudi Arabia has been conducted on this issue. Consequently, this study explored the impact of a collaboratively developed psychoeducation program delivered via a telenursing approach to determine whether it can reduce the burden on caregivers of patients with schizophrenia in Saudi Arabia.

### Study Aim and Objectives

This study evaluated the effectiveness of a collaboratively developed psychoeducation program delivered via telenursing at reducing caregiver burden among individuals caring for patients diagnosed with schizophrenia in Saudi Arabia. Specifically, the study sought to (1) identify the educational needs of caregivers; (2) assess caregivers’ perceived burden before and after the intervention; (3) compare caregiver burden scores between an intervention and control group; and (4) examine the factors associated with higher caregiver burden, such as education level, income, health status, and family support.

## 2. Materials and Methods

### 2.1. Design

To measure the caregiving burden of patients with schizophrenia, this study utilized an experimental design with two groups and pre- and post-intervention testing. This design is suitable for assessing the impact of the intervention. The period between the pre-and post-intervention testing was three months.

### 2.2. Setting

This experimental study was conducted in a tertiary governmental facility for mental health and psychiatric disorders in northern Saudi Arabia, from 1 January 2024 to 30 April 2024. The facility was built in 1983 and has a capacity of 200 beds. In 2022, approximately 40,000 patients benefited from the hospital’s services. The hospital has several departments, including admission wards for patients with mental illnesses, addictions, and forensic cases, in addition to outpatient departments, emergency rooms, and home care units. Annually, the average number of outpatients diagnosed with schizophrenia who received treatment is approximately 160 patients.

### 2.3. Sample and Sample Size

**Figure 1:**
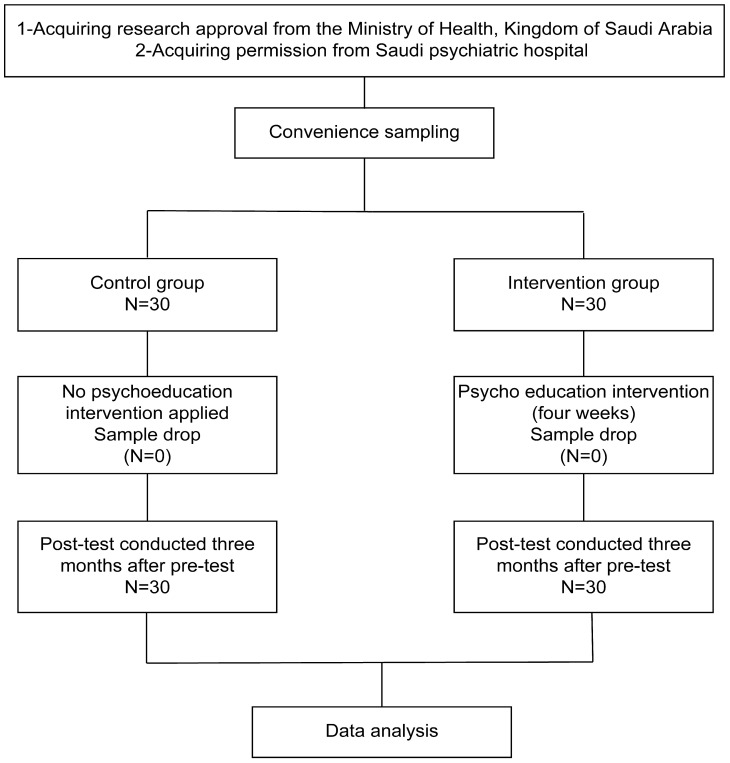
Flowchart of the research process.

The sample size was calculated by considering the mean FBIS scores values between the intervention and control groups, which were estimated to range from 1.5 to 2.0. Using a significance level of 0.05 and a power of 90%, a total of 30 participants was required in each group. Therefore, the target sample size was 60. The actual study participants were 60 families caring for patients with schizophrenia who were receiving continuous mental treatment at a psychiatric hospital. Participants were recruited using convenience sampling and then allocated into an experimental group and a control group, each comprising 30 family members caring for individuals with schizophrenia (see Figure 1).

The inclusion criteria were caregivers with no history of participating in an educational program or counseling sessions about schizophrenia; caregivers who permanently lived with and provided care to patients; and caregivers who had internet access and a phone or mobile device at home. The caregivers had to be aged 18 years or older. The exclusion criteria included caregivers with auditory or visual disabilities, behavioral and mental disorders, or those who had experienced excessive stress or emotional trauma within the six months preceding the study.

Caregivers who failed to respond to the post-intervention follow-up for three consecutive days, those who chose to withdraw from the study, and caregivers of patients diagnosed with other mental illnesses (e.g., depression or bipolar disorder) were excluded.

### 2.4. Instruments

A two-part instrument was used; the first part collected the participants’ demographic profiles while the second applied the Family Burden Interview Schedule (FBIS; see Supplementary Materials).

#### 2.4.1. Caregiver Demographic Information

To collect caregiver demographic information, a structured questionnaire was used, comprising 14 items based on relevant literature. The variables consisted of age, gender, education level, employment status, income sufficiency, household composition, smoking or substance use, family support, caregiving role, health services utilized, media literacy, and presence of physical illness or disability. Level of education was measured using an ordinal scale with four categories: (1) less than secondary education, (2) diploma, (3) bachelor’s degree, and (4) higher education (Master’s or PhD). Access to educational resources was assessed through a multiple-choice question asking caregivers to indicate their primary source of health-related information, including options such as physician, nurse, internet, books/magazines, or none. These measures allowed for a comprehensive analysis of the caregiver characteristics in relation to their perceived burden.

#### 2.4.2. Family Burden Interview Schedule (FBIS)

Caregiver burden was assessed using the FBIS, originally developed by Pai and Kapur [30]. The FBIS is a structured tool that evaluates both objective and subjective burden across multiple domains (e.g., financial strain, disruption of daily activities, and emotional stress), providing a comprehensive measure of the challenges faced by caregivers of individuals with psychiatric disorders.

The FBIS contains 26 items. The first 24 items are categorized into six categories to assess objective burden; they are rated on a three-point Likert scale: 0 = no burden, 1 = moderate burden, and 2 = severe burden. The 25th item is a supplementary item about any other family burden not mentioned in the first 24 items, and the 26th item serves as a subjective burden assessment by asking a single standard question: “How much would you say you have suffered owing to the patient’s illness?” The response to this is also rated on a 3-point Likert scale (0  =  not at all, 1  =  a little, and 2  = severely). The scores are categorized as little or no burden (0–15), mild to moderate burden (16–31), or severe burden (32–48). The total score ranges from 0–48, with higher scores indicating a higher burden.

The FBIS has been widely used in international studies involving caregivers of individuals with schizophrenia to assess the level of burden among them and has excellent validity and reliability [30]. For instance, the tool was validated in Nigeria among 368 caregivers of patients with schizophrenia in an outpatient psychiatric clinic, where the Yoruba version of the FBIS showed excellent validity with a Cronbach’s alpha of 0.830 [31]. Furthermore, studies conducted in Brazil and China confirmed the reliability and validity of the tool in diverse cultural settings, with reliability scores ranging from 0.54 to 0.90 in Brazil and an internal consistency of 0.864 in China [32,33].

We utilized an Arabic version of the FBIS that has been validated in similar Middle Eastern contexts. A seminal psychometric study involving 325 Arabic-speaking caregivers in Jordan reported a content validity index (CVI) of 0.87–1.00 (overall CVI = 0.91), and exploratory factor analysis identified four coherent burden domains explaining 74.6% of the response variance. Its internal reliability was strong, with the Cronbach’s alpha ranging from 0.87 to 0.99, and the scale effectively discriminated between high- and low-burden groups (*p* < 0.05) [34].

### 2.5. Pilot Study

To assess the clarity, feasibility, and reliability of the instruments, a pilot study was conducted with 10% of the total study sample selected at random. The pilot study also helped estimate the time required to complete the questionnaires, which was approximately 5–10 min. The internal consistency of the FBIS in this study was tested and showed a significant and positive correlation between each item and the total scale score (r = 0.92, *p* < 0.01) (r = 0.46, *p* < 0.01). The Cronbach’s alpha was between α = 0.91 and α = 0.78, confirming that the scale is reliable (Table 1). The participants in the pilot study were excluded from the main study.

### 2.6. Ethical Considerations

Ethical approval for the study was obtained from the Ministry of Health in Saudi Arabia (Approval No.: TU-077/024/243) prior to the pilot study and initiation of data collection. Participation in the study was voluntary, and confidentiality was strictly maintained by the research team. Caregivers were assured that their anonymity would be protected and that no harm or adverse consequences would result from their participation. The study’s purpose and design were clearly explained to all participants. Written informed consent was obtained from each caregiver. To ensure privacy and confidentiality, all collected data were stored on a secure, password-protected laptop accessible only to the research team.

### 2.7. Intervention

#### Development of the Psychoeducational Program

Identifying caregiver needs: The intervention process began with identifying the educational needs of caregivers to design psychoeducation programs according to their needs. A questionnaire to assess educational needs was distributed by researchers to caregivers who attend follow ups in outpatient departments with their patients with schizophrenia. The caregivers were first informed of the purpose of the questionnaire, and then they provided their responses to five questions exploring their preferences and needs regarding (1) education about schizophrenia; (2) challenges in caring for relatives with schizophrenia diagnosis; (3) attending educational programs to learn more about caring for a person with schizophrenia; and (4) preferences for modality of educational sessions, e.g., in-person or telenursing; and (5) reason for preference for educational session modality.

Review of caregiver needs: The research team analyzed the caregivers’ answers to develop the psychoeducational program. About sixty respondents indicated a need for training to care for their relatives with schizophrenia. The majority expressed a preference for telenursing due to its cost-effectiveness and accessibility, especially for those residing far from the hospital. Consequently, the program was condensed into a short, structured format.

Intervention description: The psychoeducational intervention was a structured four-week program, consisting of daily one-hour sessions delivered via Zoom—an accessible and cost-effective telenursing platform that was selected based on caregiver preference. Prior studies in Egypt and Indonesia have demonstrated the effectiveness of Zoom in delivering educational interventions in healthcare, showing its utility in increasing public health knowledge and promoting behavior change in various clinical contexts [35,36]. The intervention was specifically designed to address the distinct domains of caregiver burden identified by the FBIS, including financial strain, disruption of daily routines and leisure activities, family relationship stress, and adverse impacts on caregivers’ physical and mental health [30].

The program content addressed 16 caregiver-prioritized topics, such as the causes and theories of schizophrenia, antipsychotic medications and their complications, patient safety at home, strategies for enhancing medication adherence, managing hallucinations and delusions, suicide prevention, and coping with social stigma. Additional sessions focused on the importance of family support and the needs of caregivers themselves. These topics were selected based on a pre-intervention needs assessment and developed in alignment with best practices in psychiatric nursing and evidence-based psychoeducation [37,38]. The educational materials and session content were created by the research team through a literature review and consultation with psychiatric and mental health nursing experts. Each session employed a blended model of brief lectures, practical workshops, and facilitated group discussions to maximize caregiver engagement, knowledge retention, and peer support.

To alleviate the financial and logistical burdens, particularly for caregivers in remote or underserved areas, the intervention’s virtual format eliminated transportation expenses and reduced the time away from work. The flexible schedule allowed participants to engage without major disruption to their routines. The sessions aimed to improve caregiving efficiency through training in medication management, risk assessment, and home safety—strategies that have been shown to reduce caregiver strain and prevent health crises [39]. Simultaneously, group discussions and workshops created a psychosocial support network for caregivers, mitigating the emotional toll of caregiving and fostering shared problem solving.

Importantly, the intervention addressed culturally specific challenges such as stigma toward mental illness in Saudi Arabia, which caregivers cited as a major source of emotional burden. The sessions covering social stigma, coping strategies, and caregiver mental health helped normalize their experiences and reduce isolation—an approach supported by prior research in Saudi and Middle Eastern settings [40,41,42]. The final version of the intervention was reviewed by doctoral-trained psychiatric nurses (A.M. and L.S.) for accuracy, cultural appropriateness, and scientific rigor. Table 2 outlines the topics and structure of the intervention, while Figure 1 illustrates its implementation phases.

The final intervention was reviewed for accuracy and scientific merit by A.M and L.S., who are psychiatric nurses and hold a doctorate. Table 2 outlines the intervention topics and schedule, while Figure 1 illustrates the study implementation stages.

Intervention delivery: The intervention spanned four weeks. M.A. moderated a group discussion at the end of the fifth day of every week, which lasted for one hour (see Table 2). The educational materials were presented via PowerPoint using Zoom’s screen-sharing function. The caregivers received technological support and a training session on how to use Zoom prior to the start of the program.

### 2.8. Data Collection

The intervention process for this study involved three phases. The initial phase included the capture of baseline data. This comprised the distribution of a paper copy of the survey tool by M.A. during a clinic visit prior to the start of the educational intervention to assess the participants’ care burden. Specifically, during outpatient clinic visits, while patients were in the treatment room with their physician, the principal investigator (M.A.) provided the survey to the caregivers who were able to complete it in privacy. The second phase comprised the delivery of the four-week psychoeducation intervention by M.A. and F.R. Based on the demographic information collected during phase one, caregivers were assigned to either the intervention or control group, ensuring an equal distribution by gender, age group, and educational level. The control group received standard care (in-person outpatient clinic visits). The third phase occurred three months after the intervention and involved post-intervention data collection to reassess the caregiver burden.

The period between the pre-intervention (baseline) and post-intervention (follow-up) data collection was three months, allowing for sufficient time to assess the sustained impact of the four-week psychoeducational intervention on the caregiver burden. The same survey tool was again distributed to the participants by M.A. during clinic visits following the same procedure as in phase one. The participants received a modest financial incentive for their involvement.

### 2.9. Data Analysis

The data were analyzed using SPSS version 23.0. The frequencies, percentages, mean, and standard deviations were used to summarize the variables. An independent *t*-test was conducted to test the variance between the control and intervention groups at both the pre- and post-intervention stages. As such, in the study, a nonsignificant difference between the pre-intervention scores was a condition to validate the experiment. To examine the changes over time within the groups, a paired *t*-test was conducted to compare pre- and post-intervention scores for each group. Additionally, independent *t*-tests and one-way ANOVA were conducted to find the distribution of the FBIS scores across different risk factors. A *p*-value of less than 0.05 was considered statistically significant.

## 3. Results

### 3.1. Demographics

The control and intervention groups each comprised 30 individuals. Table 3 provides their full details including the distribution of demographic characteristics across the intervention and control groups. The participants were categorized into four age ranges, with the majority aged 41–50 years (41.67%). The sample included an equal distribution of males and females (50% each). The educational range included 50% with a diploma certificate, followed by a bachelor’s degree (21.67%), and higher education (21.67%). Employment status was classified into six categories with a predominance of employees (46.67%). Those with a less than adequate family income comprised 58.33%, with 35% living with their parents, 33.33% living with children, and 31.67% living with a spouse. Smokers accounted for 26.67%, with 10% indicating addiction. In terms of family support, 33.33% reported weak family support, 25% indicated good support (25%), 23.33% reported excellent support, and 18.33% reported moderate support. In the sample, 56.67% were parents caring for a child. Notably, 76.67% had no training, and only 35% reported a high level of media literacy. The wellness of the caregivers varied, with 45% reporting no disease and only 40% reporting good access to care support.

### 3.2. Pre-Intervention Results

**Figure 2:**
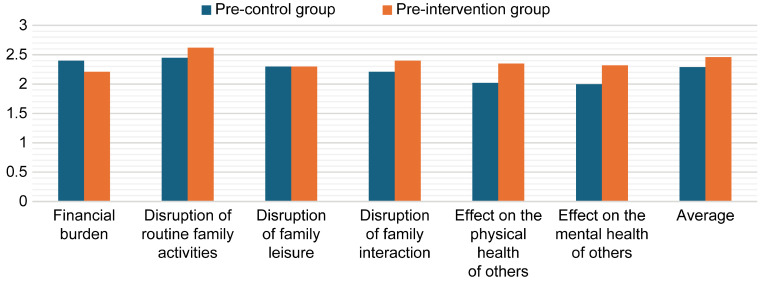
Mean pre-intervention burden scores of both groups.

An independent *t*-test was conducted to examine the differences in mean pre-intervention scores between the two groups (control and intervention), which was a prerequisite for validating the experiment. As shown in Table 4, the results indicated no statistically significant differences between the groups (*p* > 0.05), thereby confirming that baseline equivalence was achieved (Figure 2). The following section presents the post-intervention findings for both the control and intervention group.

### 3.3. Post-Intervention Results

**Figure 3:**
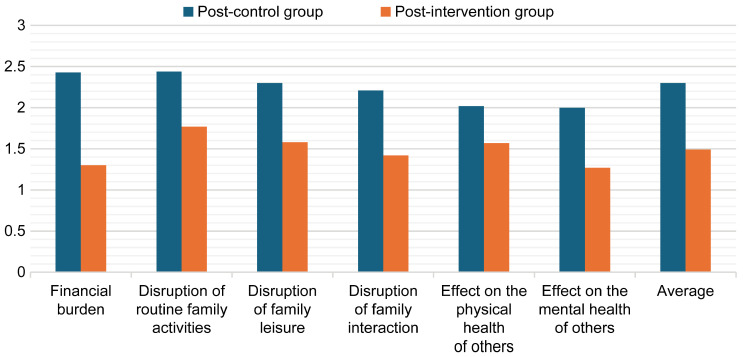
Mean post-intervention burden scores for the control and intervention groups.

An independent *t*-test was conducted to examine the differences in mean scores between the control and intervention groups at the post-intervention stage to assess the effect of the experiment, as shown in Table 5. The results revealed a statistically significant difference between the groups (*p* < 0.001), indicating that the intervention had a substantial impact. The scores decreased across all variables for the experimental group, ranging from M = 1.77 (SD = 0.33) to M = 1.27 (SD = 0.41), thereby confirming that the experiment was effective (Figure 3).

### 3.4. Control Group Results

**Figure 4:**
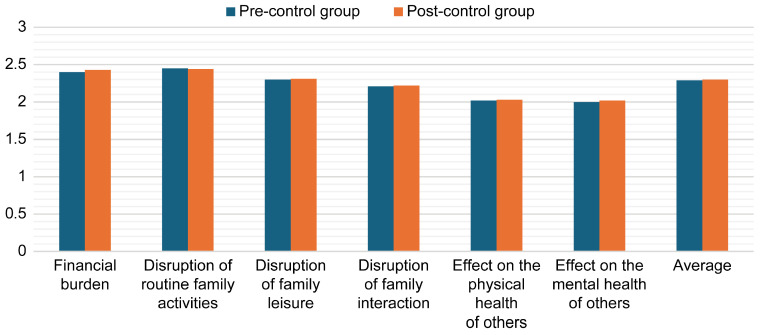
Mean pre- and post-intervention burden scores for the control group.

A paired *t*-test was conducted to examine the differences in the mean scores between the pre- and post-intervention scores in the control group. As shown in Table 6, there were no significant differences between the pre- and post-intervention scores for the control group (*p* > 0.05), confirming that there was no change in scores within this time period (Figure 4).

### 3.5. Intervention Group Results

**Figure 5:**
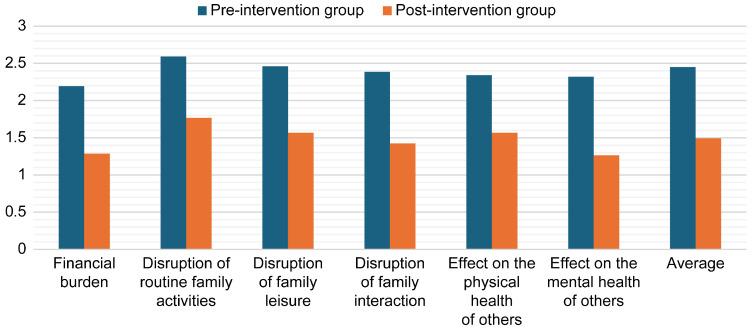
Mean burden scores in the intervention group before and after the intervention.

A paired *t*-test was conducted to examine the differences in mean pre-intervention and post-intervention scores in the intervention group at two different time points. As shown in Table 7, there were significant differences between the pre- and post-intervention scores for the intervention group (*p* < 0.001), confirming that the FBIS scores for the post-group significantly decreased (Figure 5).

An independent *t*-test and one-way ANOVA were conducted to determine the distribution of FBIS scores among those with and without risk factors in both groups before the intervention (Table 8). Four factors demonstrated a significant effect: higher education (M = 1.76, SD = 0.36, F = 20.04, *p* < 0.001), adequate family income (M = 1.83, SD = 0.57, F = 10.27, *p* < 0.001), family support M = 1.95, SD = 0.56, F = 5.08, *p* < 0.004), and caregivers with no disease were associated with a lower family burden. Lung disease was associated with the highest family burden (M = 2.91, SD = 0.19).

## 4. Discussion

This experimental study evaluated the effect of a psychoeducation program delivered via telenursing, focusing on reducing the burden on caregivers of individuals with schizophrenia. The results revealed a significant difference between the post-intervention scores of the control and intervention groups, with greater changes in the experimental group and decreased scores for all variables. This outcome is likely attributed to the content and delivery of the intervention, which was designed based on caregiver needs and preferences. The findings align with those of previous studies [39,43].

Medalia et al. [43] demonstrated that a structured psychoeducation program led by professionals trained in mental health significantly improved caregivers’ knowledge of schizophrenia, which in turn contributed to a reduction in emotional distress and caregiver burden. The role of specialized mental health nurses in facilitating the intervention is crucial, as their communication skills and clinical skills allowed for a more effective delivery of the psychoeducational material. Likewise, Uslu et al. [44] found that psychoeducation sessions facilitated by mental health nurses improved caregivers’ disease-related knowledge and promoted peer support among the participants, resulting in decreases in anxiety and caregiving strain. These findings underline the distinctive role of psychiatric nurses in engaging caregivers and providing tailored support, which may explain the greater reduction in burden observed in the intervention group in this study.

This study found that the family burden scores for the intervention group decreased post-intervention, while the mean scores for the control group (post-intervention) did not change significantly. A possible explanation for this is that initiating comprehensive psychoeducation programs through telenursing creates an opportunity for caregivers to decrease social stigma about mental illness and alleviate stress levels [44]. Addressing stigma in mental health is particularly significant in this study, as it represents a notable challenge in Saudi Arabia [40,41].

This study contributes to addressing a deficit in telehealth research by demonstrating a significant burden reduction among caregivers (*p* < 0.001). However, broader adoption demands robust economic evaluations. Previous chronic-care telehealth studies have suggested that interventions can be cost-effective—with modest incremental increases in QALYs and savings at scale—but the results are context-dependent and often require long-term follow-up [45]. Our intervention aligns with nurse-led telehomecare models, which have been shown to reduce hospitalizations and improve cost metrics in chronic-disease settings [46]. However, definitive conclusions will require embedding health economic metrics into future trials.

Challenges arise from widespread misconceptions about the nature of mental illness, such as attributing the cause to the “Evil Eye” and possession [43], alongside a lack of clinical and public-facing education [47,48]. The virtual modality of the intervention may also have contributed to the positive outcome by providing access and support to caregivers who live far from the hospital. They were supported in discussions and able to receive education about providing care for individuals with schizophrenia, while also sharing their experiences with other families. The current findings are consistent with a Turkish randomized controlled trial [49], which noted that family burden scores were higher in the experimental group before psychoeducation and there was a decrease in the total family burden score in the follow-up testing after a telenursing psychoeducation intervention. These findings, along with those of this study, add weight to the need for telenursing, demonstrating that it provides effective and more accessible support for caregivers [50].

As anticipated—and in alignment with the broader literature [51,52]—our study identified key predictors of caregiver burden, including lower educational attainment, insufficient income, poor physical health among caregivers, and limited family support. These findings underscore the need for targeted, equity-oriented interventions that address underlying socioeconomic disparities, prioritize caregiver health, and expand access to structured psychoeducation. Notably, the virtual, telenursing-based delivery model employed in this study offers a practical and scalable solution, particularly for caregivers in geographically remote or underserved areas. By reducing travel time, logistical barriers, and associated costs, this approach has the potential to serve not only as a clinical intervention but also as a health equity strategy tailored to the real-world needs of family caregivers navigating the complexities of schizophrenia care.

Our findings align with the broader international evidence showing that even minimal exposure to caregiver training—ranging from self-guided resources to formal instruction—can substantially enhance caregiver well-being and reduce perceived burdens. For instance, studies in Latin America and the Caribbean demonstrated that caregiver training was consistently associated with improved emotional resilience and caregiving outcomes, regardless of the delivery format or intensity [53,54].

Several implications arise from this study for improving support for caregivers of individuals living with schizophrenia. First, mental health hospitals should consider implementing psychoeducational programs delivered via telenursing for caregivers of patients with schizophrenia that are presented by qualified and expert nurses. These sessions should include follow-up assessments to evaluate the impact on caregiver burden over time. Collaborative approaches that incorporate the perspectives and needs of caregivers, along with aftercare services and home care, should be embedded into future interventions. Potential strategies include family and group therapy, counseling sessions, educational seminars, and physical or recreational activities to help prevent the negative effects of care burden on this population [55].

### Strengths and Limitations

While the intervention was proven effective, the study was not without limitations. The quasi-experimental design relied on self-reported data and excluded randomization, limiting the ability to determine a causal relationship between the intervention and its effects. This is an important consideration for future development and testing in different settings. Although the post-intervention scores revealed significant improvements, the study was limited by the absence of long-term follow-up to assess the sustainability of the impact. Further, caution should be taken when generalizing the findings as the sample was small and drawn from a single hospital. The relatively small sample size (N = 60) limits both the internal and external validity of the findings. Although the a priori power analysis confirmed statistical adequacy for medium effect sizes, small samples are more vulnerable to selection bias, reduced control over confounding variables, and limited generalizability to broader caregiver populations. Future studies should recruit larger, more diverse samples across multiple settings to increase the representativeness and robustness of results. Nonetheless, the findings align with those of other similar studies and may have significant potential for adaptation and application elsewhere. Importantly, the inclusion of caregivers in the development of the intervention provides a replicable mode for those seeking to tailor care to the needs of their local population.

Additionally, the use of routine care as a control condition—rather than an active or placebo-controlled comparator—may have introduced non-equivalence in engagement and attention between groups. This design decision was based on ethical and logistical considerations within the clinical context. However, it raises the possibility of Hawthorne or placebo effects, potentially inflating the observed effect size in the intervention group. While baseline equivalence was established and the control group showed no significant change over time, randomized controlled trials (RCTs) with active control arms are recommended to isolate the true effect of telenursing psychoeducation.

Further, although the intervention demonstrated statistically significant improvements across all FBIS domains, no adjustments were made for multiple comparisons. This decision was intentional due to the exploratory nature of the study and to avoid overcorrection in a small sample; however, this approach increases the potential for Type I errors. Consequently, the findings related to individual burden domains should be interpreted with caution, and future studies should apply appropriate correction methods (e.g., Bonferroni or Holm adjustments) when feasible.

Economic outcomes were not evaluated. Given the growing interest in scalable, cost-effective telehealth models, future research should incorporate economic evaluations, including cost–utility analysis, scalability modeling, and healthcare utilization metrics, to assess the broader value of telenursing interventions.

Finally, although this study focused on clinical outcomes, process evaluations were not conducted to explore implementation barriers such as digital literacy gaps, technology access in remote areas, or cultural adaptation of psychoeducational content. These factors are particularly relevant in the context of Saudi Arabia, where regional disparities in digital infrastructure and mental health stigma may influence program adoption and effectiveness.

## 5. Conclusions and Recommendations

This study provides evidence that psychoeducation delivered through telenursing significantly reduces the burden experienced by caregivers of individuals with schizophrenia. The findings highlight the effectiveness of structured, nurse-led interventions delivered remotely in enhancing caregiver well-being, especially when tailored to caregivers’ specific needs. These results support the further integration of telenursing psychoeducation as a standard component of mental health services.

Future research should evaluate the long-term effects of such interventions through randomized controlled trials and multi-site studies. Additional research is also warranted to assess the impact of telenursing on caregiver mental health, patient adherence, and hospitalization rates. Investigating the effectiveness of culturally adapted content and the influence of digital literacy in caregiver engagement may further refine the delivery and impact of telenursing programs.

## 6. Nursing Implications

The findings of this study have significant implications for nursing practice, particularly within mental health and community-based care. The successful implementation of a psychoeducation program via telenursing demonstrates that nurses can play a pivotal role in alleviating caregiver burden among families of individuals living with schizophrenia. Nurses, especially those specializing in psychiatric and mental health care, are uniquely positioned to deliver structured, evidence-based educational interventions that equip caregivers with the knowledge and skills necessary to navigate the complex demands of caregiving. This approach not only enhances caregiver well-being but also contributes to improved patient outcomes through enhanced medication adherence, effective crisis management, and increased family engagement.

Telenursing offers a flexible, cost-effective solution that addresses geographic and time-related barriers, making it especially valuable in regions with limited access to healthcare facilities. Integrating psychoeducation into routine nursing care, especially in outpatient and home-based settings, enables continuous support for caregivers and can reduce the risk of caregiver burnout. Additionally, the findings of this study support the incorporation of digital health technologies in nursing, underscoring the need for investment in professional development and to facilitate the delivery of remote care.

Moreover, nurse-led psychoeducation may help dispel the stigma associated with schizophrenia by creating a safe, informative space for caregivers to learn and share experiences. As a result, nurses can contribute not only to individual and family health but also to broader public mental health awareness. Future nursing interventions should build on this model, incorporating structured follow-up and culturally tailored content to enhance long-term effectiveness and caregiver satisfaction.

## Figures and Tables

**Table 1:** The internal consistency of the Family Burden Interview Schedule (N = 30).

Financial Burden		Disruption of Routine Family Activities		Disruption of Family Leisure		Disruption of Family Interaction		Effect on Physical Health of Others		Effect on Mental Health of Others	
No.	r	No.	r	No.	r	No.	r	No.	r	No.	r
1	0.72 **	1	0.53 **	1	0.72 **	1	0.86 **	1	0.71 **	1	0.89 **
2	0.87 **	2	0.86 **	2	0.87 **	2	0.77 **	2	0.46 **	2	0.51 **
3	0.74 **	3	0.87 **	3	0.93 **	3	0.72 **				
4	0.80 **	4	0.71 **	4	0.86 **	4	0.86 **				
5	0.86 **	5	0.92 **	5		5	0.69 **				
6	0.75 **										
α = 0.84		α = 0.91		α = 0.90		α = 0.89		α = 0.79		α = 0.78	

** *p* ≤ 0.01.

**Table 2:** Topics discussed in psychoeducation intervention.

Week	Topic	Duration	Type of Activity	
			Lecture	Workshop
Week 1	First day: Definition of schizophrenia, causes, predisposing factors, and types. Rationale: Caregivers requested that these topics be included in the psychoeducation program.	One hour	√	
	Second day: Theories about schizophrenia and treatment. Rationale: Caregivers requested that these topics be included in the psychoeducation program.	One hour	√	
	Third day: Antipsychotic medication and complications. Rationale: Caregivers requested that these topics be included in the psychoeducation program.	One hour	√	
	Fourth day: Pharmacological safety when dealing with patients with schizophrenia. Rationale: Caregivers requested that these topics be included in the psychoeducation program.	One hour	√	
	Fifth day: Group discussion. Rationale: Moderated by the research team to allow for sharing feelings and experiences among caregivers.	One hour		√
Week 2	First day: Communication skills for handling patients with schizophrenia. Rationale: Caregivers identified this topic as essential for inclusion in the psychoeducation program.	One hour	√	
	Second day: Patient safety at home. Rationale: Caregivers identified this topic as essential for inclusion in the psychoeducation program.	One hour		√
	Third day: Initial risk assessment. Rationale: Caregivers identified this topic as essential for inclusion in the psychoeducation program.	One hour	√	
	Fourth day: Factors and strategies that increase medication adherence. Rationale: Caregivers identified this topic as essential for inclusion in the psychoeducation program.	One hour	√	√
	Fifth day: Group discussion. Rationale: Moderated by the research team to allow for sharing feelings and experiences among caregivers.	One hour		
Week 3	First day: Dealing with delusions. Rationale: Caregivers requested that this topic be included in the psychoeducation program.	One hour	√	
	Second day: Dealing with hallucinations. Rationale: Caregivers requested that this topic be included in the psychoeducation program.	One hour	√	
	Third day: Suicide and suicide prevention. Rationale: Caregivers requested that this topic be included in the psychoeducation program.	One hour	√	
	Fourth day: Social stigma associated with mental illness. Rationale: Caregivers requested that this topic be included in the psychoeducation program.	One hour		√
	Fifth day: Group discussion. Rationale: Moderated by the research team to allow for sharing feelings and experiences among caregivers.	One hour		√
Week 4	First day: Complementary and alternative treatment for patients with schizophrenia. Rationale: Caregivers requested that this topic be included in the psychoeducation program.	One hour	√	
	Second day: Family support system. Rationale: Caregivers requested that this topic be included in the psychoeducation program.	One hour		√
	Third day: Needs and concerns of caregivers of patients with schizophrenia. Rationale: Caregivers requested that this topic be included in the psychoeducation program.	One hour		√
	Fourth day: Community services for caregivers of patients with schizophrenia. Rationale: Caregivers requested that this topic be included in the psychoeducation program.	One hour	√	
	Fifth day: Group discussion. Rationale: Moderated by the research team to allow for sharing feelings and experiences among caregivers.	One hour		√

**Table 3:** Caregivers’ demographic characteristics (N = 60).

	Category	Total N (%)	Intervention N (%)	Control N (%)	*p*-Value
Age group	<30 years	8 (13.33%)	4 (50%)	4 (50%)	0.997 *(χ*^2^ *= 0.035)*
	30–40 years	13 (21.67%)	6 (46.15%)	7 (53.85%)	
	41–50 years	25 (41.67%)	13 (52%)	12 (48%)	
	>50 years	14 (23.33%)	7 (50%)	7 (50%)	
Sex	Male	30 (50%)	15 (50%)	15 (50%)	1.000 *(χ*^2^ *= 0.000)*
	Female	30 (50%)	15 (50%)	15 (50%)	
Education	Diploma	30 (50%)	15 (50%)	15 (50%)	0.935 *(χ*^2^ *= 0.135)*
	Bachelor	17 (28.33%)	9 (52.94%)	8 (47.06%)	
	Higher education	13 (21.67%)	6 (46.15%)	7 (53.85%)	
Employment status	Employee	28 (46.67%)	14 (50%)	14 (50%)	0.515 *(F = 4.236)*
	Retired	5 (8.33%)	2 (40%)	3 (60%)	
	Self-employed	7 (11.67%)	5 (71.43%)	2 (28.57%)	
	Student	3 (5.00%)	1 (33.33%)	2 (66.67%)	
	Housekeeper	2 (3.33%)	1 (50%)	1 (50%)	
	Unemployed	15 (25.00%)	10 (66.67%)	5 (33.33%)	
Family income	Less than adequate	35 (58.33%)	20 (57.14%)	15 (42.86%)	0.909 *(χ*^2^ *= 0.191)*
	Adequate	15 (25.00%)	9 (60%)	6 (40%)	
	More than adequate	10 (16.67%)	6 (60%)	4 (40%)	
Cohabitants	Spouse	19 (31.67%)	10 (52.63%)	9 (47.37%)	0.139 *(χ*^2^ *= 3.954)*
	Children	20 (33.33%)	15 (75%)	5 (25%)	
	Parents	21 (35.00%)	9 (42.86%)	12 (57.14%)	
Smoking status	Yes	16 (26.67%)	5 (31.25%)	11 (68.75%)	0.190 *(F = 1.722)*
	No	44 (73.33%)	25 (56.82%)	19 (43.18%)	
Addiction	Yes	6 (10.00%)	4 (66.67%)	2 (33.33%)	0.385 *(F = 0.754)*
	No	54 (90.00%)	26 (48.15%)	28 (51.85%)	
Family support	Weak	20 (33.33%)	9 (45%)	11 (55%)	0.519 *(χ*^2^ *= 2.266)*
	Medium	11 (18.33%)	8 (72.73%)	3 (27.27%)	
	Good	15 (25.00%)	7 (46.67%)	8 (53.33%)	
	Excellent	14 (23.33%)	7 (50%)	7 (50%)	
Home caregiver	Spouse	14 (23.33%)	10 (71.43%)	4 (28.57%)	0.098 *(χ*^2^ *= 4.658)*
	Mother	12 (20.00%)	4 (33.33%)	8 (66.67%)	
	Parents	34 (56.67%)	22 (64.71%)	12 (35.29%)	
Educational resources	Doctor	5 (8.33%)	2 (40%)	3 (60%)	0.829 *(F = 1.476)*
	Nurse	3 (5.00%)	1 (33.33%)	2 (66.67%)	
	Internet	4 (6.67%)	2 (50%)	2 (50%)	
	Magazines/books	2 (3.33%)	1 (50%)	1 (50%)	
	None	46 (76.67%)	27 (58.70%)	19 (41.30%)	
Media literacy	Poor	7 (11.67%)	3 (42.86%)	4 (57.14%)	0.536 *(F = 2.167)*
	Medium	18 (30.00%)	7 (38.89%)	11 (61.11%)	
	Good	14 (23.33%)	9 (64.29%)	5 (35.71%)	
	Excellent	21 (35.00%)	11 (52.38%)	10 (47.62%)	
Disease or disabilities	Yes	33 (55.00%)	12 (36.36%)	21 (63.64%)	0.526 *(F = 0.401)*
	No	27 (45.00%)	12 (44.44%)	15 (55.56%)	
Access to care/support	Poor	21 (35.00%)	2 (9.52%)	19 (90.48%)	0.041 *(χ*^2^ *= 6.362)*
	Medium	15 (25.00%)	7 (46.67%)	8 (53.33%)	
	Good	24 (40.00%)	9 (37.5%)	15 (62.5%)	

*χ*^2^: Chi-square test; *F*: Fisher’s exact test; statistically significant at *p* < 0.05.

**Table 4:** Pre-intervention Family Burden Interview Schedule (FBIS) scores of control group and intervention group.

Variable	Control Group (N = 30)		Intervention Group (N = 30)		t	*p*-Value
	Mean	SD	Mean	SD		
Financial burden	2.40	0.72	2.21	0.68	1.07	0.28
Disruption of routine family activities	2.45	0.50	2.62	0.50	−1.35	0.18
Disruption of family leisure	2.30	0.58	2.30	0.58	−2.17	0.25
Disruption of family interaction	2.21	0.62	2.40	0.56	−2.22	0.23
Effect on the physical health of others	2.02	0.65	2.35	0.73	−1.86	0.06
Effect on the mental health of others	2.00	0.64	2.32	0.61	−1.98	0.055
Average	2.29	0.51	2.46	0.51	−1.27	0.20

**Table 5:** Post-intervention Family Burden Interview Schedule (FBIS) scores of the groups.

Variable	Control Group (N = 30)		Intervention Group (N = 30)		t	*p*-Value
	Mean	SD	Mean	SD		
Financial burden	2.43	0.68	1.30	0.45	7.61 ***	<0.001
Disruption of routine family activities	2.44	0.49	1.77	0.33	6.24 ***	<0.001
Disruption of family leisure	2.30	0.58	1.58	0.41	5.63 ***	<0.001
Disruption of family interaction	2.21	0.62	1.42	0.37	6.05 ***	<0.001
Effect on the physical health of others	2.02	0.65	1.57	0.45	3.12 ***	<0.001
Effect on the mental health of others	2.00	0.64	1.27	0.41	5.27 ***	<0.001
Average	2.30	0.50	1.49	0.30	5.27 ***	<0.001

*** ≤0.001.

**Table 6:** Pre- and post-intervention Family Burden Interview Schedule (FBIS) scores in the control group.

Variable	Pre-Intervention (N = 30)		Post-Intervention (N = 30)		t	*p*-Value
	Mean	SD	Mean	SD		
Financial burden	2.40	0.72	2.43	0.68	−0.18	0.85
Disruption of routine family activities	2.45	0.50	2.44	0.49	0.05	0.96
Disruption of family leisure	2.30	0.58	2.31	0.54	0.05	1.00
Disruption of family interaction	2.21	0.62	2.22	0.63	0.05	1.00
Effect on the physical health of others	2.02	0.65	2.03	0.67	0.05	1.00
Effect on the mental health of others	2.00	0.64	2.02	0.65	0.06	1.00
Average	2.29	0.51	2.30	0.50	−0.05	0.96

**Table 7:** Pre- and post-intervention Family Burden Interview Schedule (FBIS) scores in the experimental group.

Variable	Pre-Intervention (N = 30)		Post-Intervention (N = 30)		t	*p*-Value
	Mean	SD	Mean	SD		
Financial burden	2.21	0.68	1.30	0.45	6.06 ***	<0.001
Disruption of routine family activities	2.62	0.50	1.77	0.33	7.82 ***	<0.001
Disruption of family leisure	2.46	0.46	1.58	0.41	7.85 ***	<0.001
Disruption of family interaction	2.40	0.56	1.42	0.37	8.02 ***	<0.001
Effect on the physical health of others	2.35	0.73	1.57	0.45	4.99 ***	<0.001
Effect on the mental health of others	2.32	0.61	1.27	0.41	7.84 ***	<0.001
Average	2.46	0.51	1.49	0.30	8.96 ***	<0.001

*** ≤0.001.

**Table 8:** The distribution of Family Burden Interview Schedule (FBIS) scores in those with and without risk factors.

Factor		M	SD	Statistic	*p* Value
Age	<30 years	2.35	0.60	F = 0.63	0.60
	30−40 years	2.25	0.57		
	41−50 years	2.36	0.50		
	>50 years	2.52	0.45		
Sex	Male	2.25	0.44	t = 1.95	0.055
	Female	2.50	0.56		
Education	Diploma	2.60	0.35	F = 20.04 ***	<0.001
	Bachelor	2.44	0.50		
	Higher education	1.76	0.36		
Employment Status	Employee	2.39	0.45	F = 1.25	0.30
	Retired	2.11	0.54		
	Self-employed	2.16	0.55		
	Student	2.67	0.44		
	Housekeeper	2.96	0.06		
	Unemployed	2.39	0.61		
Family income	Less than adequate	2.56	0.38	F = 10.27 ***	<0.001
	Adequate	2.31	0.50		
	More than adequate	1.83	0.57		
Cohabitants	Spouse	2.33	0.47	F = 0.11	0.90
	Children	2.41	0.56		
	Parents	2.38	0.54		
Family support	Weak	2.46	0.37	F = 5.08 **	0.004
	Medium	2.47	0.58		
	Good	2.58	0.40		
	Excellent	1.95	0.56		
Educational resources	Doctor	2.09	0.63	F = 6.05	<0.001
	Nurse	2.33	0.58		
	Internet	1.47	0.13		
	Medical magazines and books	1.96	0.06		
	None	2.50	0.44		
Health conditions	High blood pressure	2.57	0.46	F = 3.19 *	0.014
	Heart disease	2.54	0.29		
	Diabetes	2.50	0.34		
	Kidney disease	2.55	0.52		
	Lung disease	2.91	0.19		
	No disease	2.12	0.56		

* *p* ≤ 0.05, ** *p* ≤ 0.01, *** *p* ≤ 0.001.

## Data Availability

The original contributions presented in this study are included in the article. Further inquiries can be directed to the corresponding author.

## Supplementary Materials

The following supporting information can be downloaded at https://www.mdpi.com/article/10.3390/healthcare13151922/s1, Supplementary File S1: Caregivers’ demographic information; Supplementary File S2: Family Burden Interview Schedule (FBIS).

## Abbreviations

The following abbreviations are used in this manuscript:

FBIS	Family Burden Interview Schedule
ANOVA	Analysis of Variance
