# Supplementary material for: Effectiveness of Psychoeducation via Telenursing on Reducing Caregiver Burden Among Caregivers for Patients with Schizophrenia in Saudi Arabia: A Quasi-Experimental Study
Source: Healthcare (Basel). 2025 Aug 6;13(15):1922. doi: 10.3390/healthcare13151922 (PMC12346762; doi:10.3390/healthcare13151922)
Supplement: Supplementary file 1 [file healthcare-13-01922-s001.zip › healthcare-3722499-supplementary.pdf]

Supplementary Materials

Supplementary Material S1: Caregivers' demographic information.

Dear participants,

I invite you to participate in filling out this questionnaire, which aims to evaluate the effect of psychoeducation through telenursing in reduction the burden of care givers caring for the patient with schizophrenia.....

If you agree to participate in this survey, it will take approximately 5-10 minutes to answer the survey, please answer each question and return the questionnaire at the same time to the Nursing Training Coordinator in the Nursing Department after its completion....

Thank you for your cooperation,

Participant name: ____

Signature: ____

Age: ____

○ Less than 30 years
○ From 30-40- years
○ From 40-50 years
○ More than 51 years.

Gender

○ Male
○ Female

Education

○ Diploma
○ Bachelor
○ Higher education

Work

○ Employee
○ Retired
○ Worker
○ Self-employed

○ Student

○ Housekeeper

○ Unemployed

Family income

○ More than adequate

○ Adequately

○ Less than adequate

live with

○ Spouse

○ Children

○ Parents

Smoke

○ Yes

○ No

Addiction

○ Yes

○ No

Family support

○ Weak

○ Medium

○ Good

○ Excellent

Home caregiver

○ Spouse

○ Father

○ Mother

○ Parents

Educator resources

○ Doctor

○ Nurse

○ Family

- Relatives
- Internet
- Medical magazines and books
- Friends
- No training

Media literacy

- Weak
- Medium
- Good
- Excellent

Having disease or disabilities

- High blood pressure
- Heart disease
- Diabetes
- Kidney disease
- Lung disease
- No disease

Access to care and support

- Weak
- Medium
- Good
- Excellent

Supplementary Material S2: Family burden interview schedule (FBIS).

Instruction: interview the relatives in the following guidelines and note your rating for each general category, as well as for everyone's items on a three-point scale.

0: No burden **1: Moderate burden** **2: Sever Burden**

	**A. Financial burden**	**0**	**1**	**2**
1	Loss of patient income and its effect on family income			
2	Loss of income of any other member due to patients			
3	Expenditure incurred due to patients and treatment and its effect on family finances			
4	Expenditure incurred due to extra arrangements			
5	Loans taken, their effect on family finance and savings spent			
6	Any other planned activity put off because of financial pressure owing to patient illness			
	**B. Disruption of routine family activities**			
7	Patients do not go to school, college, work etc.…			
8	Patients not helping in household work			
9	Disruption of activities of other family member			
10	Patient behavior disrupting activities			
11	Neglect of the rest of the family due to patient illness			
	**C. Disruption of family leisure**			
12	Stopping of normal recreational activities			
13	Patient illness using up another person's holiday / leisure time			
14	Patient lack of attention to other members-children and its effect on him			
15	Any other leisure activity had to be abandoned due to patient illness			
	**D. Disruption of family interaction**			
16	Any ill effect on the general atmosphere in the house?			
17	Do other members get into arguments?			

18	Have relatives and neighbors stopped visiting the family	
19	Has the patient's illness had any effect on relationship?	
20	Has the family become secluded?	
**E. Effect on physical health of others**		
21	Have any other members suffered physical ill health?	
22	Has there been any other adverse effect on health?	
**F. Effect on the mental health of others**		
23	Has any other family members sought help for psychological illness?	
24	Has any other family members lost sleep, depressed, irritable	
**G. Subjective burden on the family**		
25	How severely would you say you have suffered due to the patient's illness?	
26	Are there any other family burden not mentioned in the questionnaire?	

<u>The scoring system includes:</u>

No burden or little from 0-15

Mild to moderate burden from 16-31

Severe burden from 32-48
